# Extracts and Gleanings

**Published:** 1871-07

**Authors:** 


### PART II.

### Abridged Extracts and Gleanings from our Exchanges

*MULTUM IN PARVO.*

Epilepsy—Injection of Morphia.—In the spanæmic condition of brain found in epileptics, morphia injected subcutaneously acts like a charm, subduing the pain of epileptic neuralgia, and sometimes postponing, if not preventing, a convulsive seizure.—Dr. E. T. Wilson.

Hypodermic Syringe.—Dr. A. J. Perkins says the hypodermic syringe is an effectual instrument in the cure of those encysted tumors of the scalp usually called wens, where the patient objects to the knife. Charge it with pure tincture of iodine and insert the point well into the body of the tumor, and force in as much of the tincture as the tumor will contain. In a few days the sac shrinks away from the surrounding parts and may be easily removed. The syringe forms the best exploring needle yet made, as a syringe full of the contents of a swelling can be drawn out and examined at leisure.

On the Local Application of Ice and Cold Water in Scarlatina, Diphtheria, Croup, etc.—Dr. Corson, of Pennsylvania, in a series of able papers, calls (Philadelphia Med. and Surg. Rep.) attention to the happy results accruing from the use of the above agents in scarlatina, diphtheria, croup, quinsy, etc. In his own practice, and that of many others who had given these agents a fair trial, ice-water applications to the throat externally, and ice in substance as a gargle to the throat, have been attended by almost magical effects. Fatality was vastly lessened, recovery was rapid, and sequalæ were uncommon. His cases of scarlatina and those of others mentioned were not selected, but all were given—malignant, anginose and simple. Dr. C's observations as to the effects of these agents run through some twenty or more years. As his experience with them has increased, so has his faith. Dr. C. reasons that the diseases in question are originally local in their action, the systemic trouble being secondary. Combat and arrest the local trouble, and the systemic disorder will be prevented or lessened. He condemns the practice which consists in caustics, astringents, irritants, and poultices to the throat and neck, as but adding fuel to the fire. The indications are to cool the burning throat, and constringe and tone up the blood vessels, and lessen the fever. All this is accomplished by allowing the patient to suck ice, to gargle the throat with ice water, and by enveloping the neck in bags of ice, and by cold affusions to the general surface. This practice is to be kept up steadily throughout the whole course of scarlatina. This, together with support by nourishment, is all that need be done in the mildest as well as the severest cases. Albuminuria and enlarged glands are no contra-indications to the use of ice and ice-water. Dr. C. contends that there is no danger of retrocession of the disease under the use of these agents. He gives over one hundred cases of scarlatina in one year, all treated by these means, with not a single fatal result.

The same treatment is applicable to diphtheria, membranous croup, and quinsy. Dr C. has observed the happiest result from it in these affections. He expresses surprise that our text-books on practice and the diseases of children have given hardly a passing notice to these simple therapeutics in the management of diseases so dangerous to life.

*New uses of Arnica Montana.*—The Journal of de Chinie Medicale, announces three new applications for Arnica Montana. 1st, for painful hemorrhoidal tumors; the second for varicose veins of pregnant women, and the third in cases of tremors occurring in gilders, amauretic amblomynia.

The alcoholic tincture of Arnica, from the fresh juice, diluted with four parts of water, always relieves hemorrhoidal tumors.

M. Liebeck administers the Arnica internally to pregnant females in varicose veins. He infuses one gramme of the flowers in 250 grammes of water, and adds to it a few grammes of alcohol and gin, a table-spoonful four times a day, which reduces the pains, and makes the enlargement gradually disappear.

M. Thalmann, of St. Petersburg, prescribes, in the affections common to gilders an infusion of 12 grammes of the flowers of arnica in 200 grammes of water, of which a table-spoonful is given every two hours.

TURPENTINE IN PUPURA HEMORRHAGIA.—Dr. Nelegan says it acts as a powerful cathartic, having the power of checking the hemorrhage. I prescribed the oil, both in the form of a draught and of enema—the usual dose for an adult being from one ounce to an ounce and a half, and for children from two drams to half an ounce, generally in combination with castor-oil, to render its cathartic action more certain, since which time I have used it with decided benefit.

On the treament of Scrofulous Diseases.—By M. Landrat.—He first puts his patient upon generous diet, consisting of roast or fried meats, bread aud vegetables, with wine and water, and exercise in the open air. He uses three sorts of baths, gelatinous, intended to soften the skin, alkaline and sulphurous. The alkaline are made of soda or ordinary soap. When a greater degree of stimulation is required, in cases of flabby ulcerations, he uses sulphurous baths. In addition to baths, he makes use of three principal internal medicines, iodine, extract of walnut and steel. Iodine is given under different forms, as tincture, iodide of potassa, in simple solution, in pills of the iodide of iron, or as the proto-iodide of mercury, or as it is found in cod-liver oil. He recommends the doses to be small, as the tincture of iodine, the ioduretted hydriodate, and the iodide of iron, too readily decomposes; the cod-liver oil he finds too nauseous; the iodide of potassium, therefore, in solution, either simple or with the addition of iodide, is the formula which he generally prefers. Indeed, he thinks that he is now able to replace iodine with a medicine equally efficacious, and more easy of administration. The extract of green walnuts, in the form of a syrup, in tea-spoonful doses several times a day. The appetite is increased, digestion is improved, and the scrofulous aspect is gradually dissipated.

Treatment of Chorea.—M. Favre de'Esnans, says in the London Lancet, that he has obtained the happiest effects in epilepsy and chorea, from the use of prussiate of iron. The following is his formulary:

| | | |
|---|---|---|
| Prussiate of iron, | . . . | grs. xv. |
| Extract of valerian, | . . . | grs. xxxv. |

Make 24 pills, and give one pill every six hours, each pill to be followed by a wineglass of infusion of valerian.

Treatment of Leucorrhœa.—Dr. Osborne, of Alabama, has found the following uniformly successful:

To 2 ounces of a decoction of red oak bark, add 10 drops of the muriated tincture of iron, and inject into the vagina two or three times daily.

Vomiting of Pregnant Women.—Dr. Stackler has reported that the black oxide of mercury given in doses of three-fourths of a grain daily, is effectual in arresting the obstinate vomiting of pregnant women, and said to be equally appropriate in irritated states of the uterus, whether in pregnancy or otherwise.

Croup is said to be successfully treated in its secondary stages by administering in doses of one fourth to a half grain every half hour, until free emesis be induced.

Coffee as a Powerful Antidote.—Dr. Max Langenschwarz in an article from the American Medical Gazette and Journal of Health, takes the position that coffee is a successful antidote of 53 of the most powerful poisons, by the use of the "tincture of coffee," made as follows:

Take a quarter of a pound of green coffee (common Domingo the best,) and boil it with one quart of water, till it is reduced to one pint; then put in a bottle and add one quart of alcohol, and shake it from time to time, which gets stronger, and will retain its strength for years, if kept stopped. If you will add to the alcohol about two table spoonsful of spirits of camphor, you will double and triple the anti-poisenous quality of the tincture. To be administered naturally and by clyster—the internal dose about ten to twelve drops in a teaspoonful of water every five minutes, and every fifteen minutes when the patient begins to recover. Large, and even very large doses may be given, if the danger of life is imminent.

Radical Cure of Hydrocele.—Dr. A. A. Harvey, in the London Lancet, has cured this disease by first discharging the fluid with a trocur or lancet, and then apply a vinegar poultice over the scrotum, in order to bring on inflammation, which takes place in a few hours, and becomes painful. When sufficient inflammation has been excited, remove the vinegar poultice and apply a milk and bread poultice. In a short time the pain and inflammation generally subsides, and the cure is complete. Give a few doses of purgative medicine.

New Mode of Inducing Vomiting.—The New Hampshire Journal of Medicine reports a case of poisoning by opium, in which, after the failure of the usual means for producing vomiting, the patient was made to swallow two glasses of vinegar and water immediately followed by ʒii. of carb. potassa in water. A powerful effervescence took place, which instantly produced copious vomiting.

Chloroform Ointment.—Mr. Curling recommends the following as highly beneficial in irritable ulcer of the rectum; Take chloroform, one fluid drachm; oxide of zinc, half drachm; olive oil, one fluid drachm; simple cerate, four drachms. Mix.

Gangrenous Ulcers.—A poultice formed of yeast and wheat bran is valuable. It tends to destroy the fetor, arrest the sloughing, assists in the separation of the dead parts, and establishes a healthy granulating surface.

Senile Gangrene.—The internal use of muriate of ammonia and local applications, in form of fomentations and pediluvia, of a strong solution of this same salt are often most advantageous.

Citrate of Caffein in Neuralgia.—Dr G. W. Arnett, of Bossier Parish, Louisiana, reports a number of cases in which he has had great success in the treatment of neuralgia, by the use of citrate of caffein and sul. morphia; also in nervous headache, hysteria, and other similar diseases. His prescription varies in amount to suit the case, the average being—

| | | |
|---|---|---|
| Sul. morphia | . . . . | grs. ½. |
| Caffein, | . . . . | grs. iij. |
| Citric acid, | . . . . | grs. iij., |

to be given in some warm coffee, or what is better in a decoction of race ginger. The caffein and citric acid will, in the majority of cases, relieve nervous irritation without the addition of the morphia, which is a desideratum when the bowels are constipated. It acts powerfully on the skin, equalizes the circulation, and thereby removes local congestion.

Chionanthus Virginica.—A small tree, or shrub, commonly known as the Fringe tree, growing from Pennsylvania to Georgia, presenting clusters of snow-white flowers in May and June, and from its white appearance, has received the name of "old man's beard." It is said to possess tonic, alterative, anti-periodic, diuretic and aperient properties. The most important therapeutical property, is its specific power over morbid conditions of the liver. It is as much a specific in jaundice as quinine in periodical fevers; and cases of years' standing may be removed in from eight to ten days. The mode in which it has been used is to take a tincture of the bark of the root in gin, say two ounces to a quart of gin, and give half an ounce of this every three hours; or the fluid extract, and give from one to two drachms every three hours.

Bromide of Potassium and Ammonium in Insomnia.—In the Reports of New York Academy of Medicine, Oct. 3d, 1866, a case of delirium tremens is instanced, which was successfully managed by the use of the following formula, after a failure of valerianate of zinc and certain other well known anti-spasmodics to control the violent manifestations.

Take potass. bromid; ammon. bromid; of each one and a half drachms; aqua distil., one ounce; dissolve; let him take a spoonful at bed time, as often as occasion may require.

Dr. Garrish alludes to a case where the insomnia was effectually subdued by the bromide of potassium alone. Dr. Bulkley speaks highly of the salts in combination.—*Medical Record.*

Chloride of Zinc Paste.—Take chloride of zinc, two drachms; sanguinaria, powdered, one drachm; pulv. acacia, two drachms. Mix; add sufficient water to form paste.

On the use of Arsenic in Certain Painful Affections of the Stomach and Bowels.—Dr. Arthur Leared, in an article on this subject in the Medical Times and Gazette (July 23, 1870), states that pain after food is a very common symptom of dyspepsia, and in many cases seems to constitute the disease. "This pain usually yields to medical treatment and proper diet. But there is another kind of gastric pain far more severe than that which depends on food, and which does not yield to ordinary remedies. I have elsewhere pointed out how this pain may be removed, and the subject seems of sufficient importance for some further remarks.

The curative effects of arsenic are most striking in severe cases of paroxysmal pain, and its success becomes doubtful in proportion as the case assimilates to those in which a lower degree of pain is traceable to the influence of food. In determining the question of the fitness of a case for the arsenical treatment certain circumstances may render essential aid. If the disease came on after some mental shock or severe trial, if the patient has previously unmistakably suffered from neuralgia, if he has lived in a marshy district, and especially if he has had hemicrania or ague, and if, in addition to the occurrence of one or more of these circumstances, the pain is paroxysmal, it will almost certainly yield to arsenic. But as already said, there are other cases suitable for the treatment, and they are the most numerous, in which the pain closely resembles that which attends dyspepsia It is sometimes extremely difficult to make the diagnosis between neuralgic pain of the stomach or bowels and the pain caused by gall-stones. But in my previous paper I have gone into details respecting this and other sources of error.

A few words will suffice as to the particular preparation of arsenic to be selected, and the extent to which it should be used. In most cases the liquor arsenicalis answers every purpose, but when the system is more than usually susceptible of the action of the mineral, the liquor sodæ arseniatis seems to irritate less, and in a few instances the acid solution of arsenic is to be preferred to either. Whatever preparation be selected it should always be taken immediately after food and, notwithstanding that its beneficial action may have been previously observed, it will be proper to continue the medicine until its constitutional effects are well marked. Notwithstanding what has been said to the contrary, I do not believe that the proper use of arsenic as a medicine is followed by any injury to the system.

Bromide of Potassium in Hydrophobia.—A marked case of this terrible disease, is reported to have been successfully treated with large doses of bromide of potassium.

Lactate of Iron.—Mr. James C. Dickinson extols (Lancet, Aug. 20, 1870) the use of this remedy in anæmia, general debility, leucorrhœa, hypertrophy of the spleen, for boils, and as a prophylactic against malarious fevers. He says, "it possesses all the properties of the other preparations of iron; as a blood-restorer it is a very effectual remedy; it does not produce constipation, is almost devoid of any disagreeable taste, and in most cases rapidly restores the appetite. As it possesses scarcely any astringency, it may be given when the stomach will not bear the more styptic preparations of iron.

"The dose for an adult is a teaspoonful gradually increased to two, taken either in water or soda-water; and for children half a teaspoonful twice a day, and gradually increased according to age."

Urethral Stricture.— Prof. Mitscherlich uses the hair from horse-tail, by means of which he masters every case. Notwithstanding its superior fineness to that of the best bougies, this horse hair possesses an elasticity and a solidity sufficient to make one careful in its introduction lest he tear the walls of the canal or of the bladder. After having well oiled the urethra he introduces them the same as if they were bougies. The most narrow points have been passed by this method. M. Mitscherlich has even obtained consecutive dilatation of the strictured point by means of these same horse hair, either uniting two, three or more of them into a pencil by means of an elastic glue, and in such a manner as to form a regularly ascending scale, or, using the horse hair as a conductor, over which he passes hollow bougies open at each extremity.

Calabar Bean in Suppuration of the Cornea.—M. Galezowski expresses himself (Annaire de Therapeutique, 1870.) in favor of the instillation of solution of calabar bean in the treatment of this form of ophthalmic disease. The contractile action exerted by the bean on the vessels of the cornea opposes dilatation and congestion, and singularly aids the cicatrization of wounds. Belladonna, which produces opposite effects, should, he thinks, be discarded in these affections.

Galactorrhea.—The excessive flow of milk has been diminished by the use of ergot. Dr. Le Gindre has been successful in several cases of enormous lacteal secretion, by the following potion, which effected the desired result in from 9 to 12 days; take of ergotine of Borjean 30 grains, syrup 1 fluid ounce, water or other vehicle 4 fluid ounces; mix. The dose is a tablespoonful, three times a day.

CARBOLIC ACID IN PHTHISIS.—Prof. Longet and Drs. Wolfe and Labort, have administered this acid in numerous cases, in the different stages of phthisis, with great benefit. Fifteen drops of the pure acid are dissolved in two drachms of spirit, and the solution mixed with thirty-two ounces of water, and this quantity was administered daily by the stomach, and partly by the inhalation of the fluid in a pulverized state.

DIGITALIS IN PNEUMONIA.—Dr. Gallard, of the Hotel de la Pitie, relates a case of a young man who was cured by the use of digitalis, after a most unsuccessful and barbârous treatment by blistering, cupping, tartarized antimony, etc., which was discontinued, owing to the great weakness and depression which it produces.

ANTISPASMODIC MIXTURE.—Chloroform, two and a half drachms; essence of orange peel, twelve and a half grains; essence of bitter almonds, seven and three fourth grains; alcohol, at 90 degrees, ten ounces; sugar, four ounces. Water, sufficient to make one quart. Dose, one to two glasses.

POTION FOR DIARRHEA.—Syrup of rhatany, ten drachms; tincture of catechu, half an ounce; carbonate of lime, one and one-fourth drachms; laudanum, twenty-five drops; peppermint water, five ounces. A teaspoonful every half hour.

BELLADONNA AS A LOCAL APPLICATION.—A belladonna plaster applied over the loins is said to arrest diabetes insipidus. In painful uterine affections it is often found valuable if applied over the sacrum.

CARBOLIC ACID AS AN INHALATION.—A solution of one grain of carbolic acid to four ounces of water, of which a small quantity should be inhaled frequently, is very beneficial in asthenic laryngitis and bronchitis.

NERVOUS APHONIA.—A case of nervous aphonia is reported to have been cured by the daily administration of one-sixteenth of a grain of atropia.

PRURITUS PUDENDI.—The following is a most excellent application: Take sodæ sulphis, one drachm; water, three drachms; glycerine, one ounce; mix.

LINIMENT FOR SPRAINS, LAMENESS AND BRUISES.—Two ounces of oil of spike; two ounces of origanum; two ounces of hemlock; two ounces of wormwood; four ounces of sweet oil; two ounces of spirits of ammonia; two ounces of gum camphor; two ounces of spirits of turpentine. Add one quart of proof spirits, 95 per cent; mix well together and bottle tight. Try it.

IODINE.—Inquiry is often made regarding the composition of the "iodine caustic," and the "compound tincture of iodine," of Churchill. By the kindness of Dr William Neergaard, we are able to give the formula as follows: Churchill's iodine caustic is composed of "iodine, one drachm; iodide of potassium, two drachms; water, half a fluidounce; mix." This mixture will be seen to be more than five times the strength of liq iodinii co. of the U. S. Pharmacopœia, which it otherwise resembles. Churchill's compound tincture of iodine is: "pure iodine, two and a-half ounces; iodide of potassium, half an ounce; alcohol, four fluidounces; rectified spirit of wine, twelve fluidounces; mix them." Each fluidounce of this tincture contains seventy-five grains of iodine, five times as much as the tincture bearing the same name in the U. S. Pharmacopœea.—*N. Y. Med. Journal.*

IODINE IN INCONTINENCE OF URINE IN OLD PEOPLE.—Dr. Schmidt, of Munsterfield, having witnessed useful effects from the exhibition of iodine in incontinence of urine resulting from paralysis, determined to try it in other cases. An old lady, aged eighty, who had always enjoyed good health, and was very active for her years, was at acked at the age of seventy-six with dysentery, which very much weakened her. From this time the urine passed involuntarily, and for four years she suffered great misery in consequence; from her age her condition was looked upon as incurable. The author gave her one drop of tincture of iodine every hour, and the following day she was able to hold the urine, and she continued the medicine (every two hours one drop) for a fortnight, and with complete success. The discontinuation of the medicine for some time led to a return of the symptoms, which disappeared, however, directly the medicine was resumed. It was continued, therefore, with occasional suspension, for two years, when she died from the effects of a blow.

Another case was an old man, aged seventy-four, who had suffered for six months from the same affection. He was ordered pills, containing each $\frac{1}{10}$ grain of iodine. Immediate improvement followed; he was never quite able to do without the remedy. He died eighteen months later, from inflammation of the lungs.—*Ibid.*

COMP. CH. PECTORAL.—This invaluable compound will be found to meet the approval of medical men, everywhere, when fully tested.

℞—Acet. Morph, . . . grs. iv.

Tinc. Sang. Can., . . ʒ ij.

Vin. Antim. Tartart.

Vin. Ipecac, . . . *aa* ʒ iij,

Syrup Pruni Virg. . . ℥ iij. m.

SLEEPLESSNESS DURING FEVER.—The most important medical property of Salves is the application of the moistened leaves to the bare scalp in severe cases of fever, attended by delirium. If it succeeds in inducing sleep under these circumstances, it will be an invaluable remedy, for we know of no more deplorable condition, or one more fraught with danger, being the fore-runner of death. A leaf of Tobacco, if often applied over the radial artery, or the pulse at the wrist, seldom fails of producing vomiting.—*Quarterly Medical Journal.*

THE USE OF CAMPHOR IN HOSPITAL GANGRENE—M. Netter, of the Military Hospital of Rennes, has found camphor, in powder, very efficacious in *hospital gangrene*, by sprinkling it abundantly over the wound In his service as well as in that of M. Aubry, surgeon of the same institution, they had treated this affection by the usual means—chloride of iron and carbolic acid with alcohol —but without success. By using very freely the powdered camphor, three patients were successfully treated, and in forty-eight hours the disease disappeared from the hospital.

The *Medical Times and Gazette* (September 3, and October 1, 1870) contains articles commendatory of the use of iodine (two grains of iodine and a little iodide of potassium to an ounce of water) as a topical application to wounds, of its use in syphilis as a gargle, and in form of an ointment externally to relieve the asteocopic pains in the skin-bones.—*N. Y. Med. Journal.*

IODURET OF SILVER IN WHOOPING-COUGH.— Dr. Bartlett (*Am. Practitioner*, February, 1870) recommends the ioduret of silver as a remedy for whooping-cough. He prepares it by adding a solution of iodide of potassium (one drachm to the ounce) to a solution of nitrate of silver (a drachm to the ounce). The yellow precipitate is well washed till the nitrate of potash is removed. For administration Dr. B. used the following formula: ℞. Arg. iod. ten grains, sacch. alb. seventy grains, pulv. gum. tragac. ten grains. Rub well together, moisten with a few drops of water, and make of the mass eighty pills. A child two or three years old should take one pill three to five times daily, just before or after meals. Children from six to ten years should take two pills for a dose. The Doctor thinks that any one who will try the remedy for a week will be satisfied of its great power in diminishing the paroxysms of the cough.—*Ibid*

ON THE TREATMENT OF VENEREAL VEGETATION.—It is said that a powder composed of equal parts of burnt Alum and powdered savine, will destroy the vegetations immediately. Two applications in the day are sufficient.

*The Use of the Bath in Fever.*—Dr. Fahrsen (*Lancet* December 31, 1870), writing from Dresden, reports equally good results from the same treatment in the hospital for fever patients sent from the front. Some portion of these patients in this hospital were women. Dr. Fahrsen saw 200 cases treated, with a mortality of four per cent.

In taking the temperature, the thermometer was placed in the anus in all cases, except the women, in whom auxillary temperature was taken—the anal temperature being much more speedily and certainly obtained.

The use of water, chiefly in the form of a cold pack, has been revived in scarlatina (*Lancet*, July 16, 1870) and in relapsing fever (*Edenburgh Medical Journal* July, 1870), with very considerable success.

We may mention in this connection the use of *salicine* in typhoid fever, from which great benefit is said to be derived. The curative action is dependent, it is claimed, upon the antiseptic power of the drug.—*Ibid.*

The following recipes are from the private prescription-book of an old and experience physician, and given for what they are worth:

CHILL AND FEVER.—

| | |
|---|---|
| Sul. Quinine, . . . | grs. 60. |
| Muriated Tinc. Ferri, . . | ℥ iss. |
| Liq Arsenicalis, . . . | ♏. 160. |
| Sul. Acid, . . . . | ♏ 15. |
| Aqua Puræ, . . . | O. 1. m. |

Teaspoonfull three times a day.

FOR DYSMENORRHŒA.—Dr. E. D. Fenner, M. D., for a number of years, has used for this disease the following:

| | |
|---|---|
| ℞—Gum Guiac, . . . | ℥ i. |
| Balsam Canada, . . . | ℥ i. |
| Oleum Sarsafras, . . . | ʒ ij. |
| Corro. Sublimate, . . . | grs. xx. |
| Alcohol, . . . . | ℥ viij. m. |

Digest the Guiac and Balsam several days in half the amount, and the Mercury in the other—then combine the two.

DOSE.—10 to 20 drops night and morning, in a glass of water. Dr. F. begins a day or two before, and gives 25 drops, in sage tea, night and morning, until the discharge ceases, then discontinue until the next period. In severe cases commence ten days before. If the pain should be severe at the time, give

| | |
|---|---|
| Spts. Camphor, . . . | ʒ iij. |
| Chloroform, . . . . | ʒ ij. |
| Tinc. Opii, . . . . | ʒ j. |

Sig—Teaspoonfull every hour.

Whooping-Cough.—

| | |
|---|---|
| Alum, . . . . . | grs. xxv. |
| Syrup of Morphia, . . . . | ʒ ij. |
| Ext. Conii, . . . . | grs. xij. |
| Aqua Menth, . . . . | ℥ iij. m. |

Dose.—Teaspoonfull every or four hours.

To Remove Stains of Silver.—Dr. Parsons, of Bristol, England, proposes to remove stains of nitrate of silver, by a solution of corrosive sublimate in muriate of (hydrochlorate) ammonia.

Dr. Heberden's Treatment of Dysentery.—Saline Purgatives, with small portions of Ipecacuanaha. To adults I have uniformly given of Sulphate of Magnesia, one drachm combined with a grain of Ipecac. in some simple aromatic water, every six hours; children, about half the quantity. So soon as the natural fecal dejections are produced, the bloody mucosties cease to be discharged, the tenesmus disappears, and the patient is cured.

Internal Use of Nitrate of Silver in obstinate diarrhœa and dysentery. Dr. Thomas Aikin, says: "When the lower portion of the intestinal tract was presumed to be the seat of ulceration, enemata, containing from one to three grains dissolved in distilled water, were administered. In most cases one enema sufficed. In other cases, the remedy was given by the mouth, in half grain doses every half hour, formed into pills, with tragacanth or starch, until, from two to four grains were thus taken. In some instances these two modes were combined; the results were that only two cases of the fifty cases "succumbed."

Muriated Tincture of Iron in Erysiplas.—In the first instance have the bowels freely acted upon. If the erysiplas is mild, administer 15 drops of the Tincture of Iron in water, every two hours, until the disease is completely removed. When more severe give 25 drops every two hours, for several nights and days, however high the fever and delirium. The local applications I ever find necessary to use, are hair-powder and cotton-wadding. —*G. Hamilton Bell.*

Exanthemic Liniment.—

| | |
|---|---|
| ℞—Ol. Crot., . . . | xx m |
| Antim. Tart., . . . . | ℈ i. |
| Liq. Potassa . . . . . | ℥ i. |
| Aqua Puræ, . . . . | ℥ vii. m. |

—*Dr. Morris.*

Digitalis in Earache.—A correspondent recommends the introduction into the meatus of a piece of cotton saturated with tincture of digitalis.

M. Velpeaus' New Caustic.—He has found that sulphuric acid, with saffron, forms an admirable caustic. The powerful counterizing effects of the acid are not destroyed, but by the combination, a black paste is formed, which hardens into a crust alter expo ure to the air. Its peculiar properties consists in its great power. with facility of circumscribing its action, and the eschar formed, through deep, is quickly thrown off. In cancerous affec·tions, the peculiar and disgusting fetid odor was quickly destroyed, nor did any bad effects indicating its absorption, arise during its use.

Astringent or Anti-Diarrhœa Medicine.—The following prescription has been used for the last ten or fifteen years, by Dr. Dugas, in diarrhœa and cholera morbus with such marked success, that he has given it to the public:

Tinct. Catechu, two parts.

Tinct. Opii.

Tinct. Camphor.

Tinct. Myrrohæ.

Tinct. Capsin, of each one part.

*Mix*—Dose, from one to two teaspoonsfull.

Chloral Hydrate in Nocturnal Enuresis.—Dr. J. B. Bradbury states that he has used the chloral hydrate successfully in the treatment of this affection. He mentions one case particularly, a girl fifteen years of age, who had wetted her bed every night for nine years. She was ordered fifteen grains of the chloral every night, and after taking the first dose there was no return of the complaint. She had no relapse at the end of six weeks. Other cases are given showing the marked beneficial results from the use of the agent in this affection. He claims that chloral possesses the following advantages over belladonna: I. The effect of belladonna is not so immediate, frequently taking weeks to produce any marked control over the disease; whereas the influence of chloral hydrate is most rapid, the malady frequently diappearing after the first dose of the remedy. This quick improvement cannot be over-estimated in the treatment of these affections, upon which the mind exerts a powerful influence. 2. That belladonna sometimes induces profuse diarrhœa. 3. That belladona, when pushed to the extent to which it is necessary to be really efficacious, not unfreqently impirs vision, etc., which is not the case with chloral hydrate. 4. That belladonna has often failed to be of any service.—*Am. Pract.*

Excessive Typanitic Distention may be relieved by a clyster of cold water—cold contracts the bowels, and diminishes the expansion of the gass, while the water at the same time absorbs a portion of it. This injection may be frequently repeated.—*Medico Chirurg Review.*

Iodide of Potassium in Bright's Disease.—Prof. Crequi, of Brussels. has given iodide of potassium with good result in the second stage of Bright's disease. He says that those who have previously tried this remedy have failed because the doses have not been sufficiently large He begins by giving from fifteen to to forty-five grains daily, increasing the daily quantity by fifteen to twenty grains, until an amount varying from seven to fifteen grammes (or even more, if the symptoms appear to demand it) is reached. Favorable results from the use of the iodide in severe albuminuria have also been noticed by Dr. Brandon and Dr. J. Semmalla, of Naples. Dr. Caspari, of Meiningen, has (*Deutsche Klinik*, Nov. 27, 1870) given the iodide of potassium in five cases. A good result followed in three: the urine became free from albumen, the dropsy disappeared, and the patients regained strength. The other two patients died. The explanation offered of the action of the iodide of potassium in Bright's disease is that it diminishes the exaggerated productivity of the connective tissue, which is manifested in parenchymatous nephritis by the production of spindle-cells around the Malpighian bodies.—*Wiener Med. Wochenschrift.*

Bromide of Potassium in Uterine Fibroids.—In the Boston Gynæcological Society a case of fibroid tumer of the uterus, which had been as large as a "small child," was reported cured by the steady employment of bromide of potassium for eighteen months. Dr. Storer had used both this salt and the chloride of ammonium—the latter in some cases with benefit, though he seemed to think that the occasional shrinkage and disappearance of such tumors, previous to the passage of the climacteric, were rather coincidences than the effects of treatment. Dr. Sullivan preferred the bromide to iodide, because it "produced a more profound effect on the nervous system, and hastened molecular metamorphosis."—*Pacific Medical Journal.*

Styptic Colloid, for promoting the healing of wounds by the first intention, or for treating open and fœtid wounds, and for are resting hemorrhage, is well known to the profession. We have found the colloid a very good application for herpes zoster. Mr. Browne (*Liverpool Hospital Reports*, vol. iv.) recommends us to paint a patch of eczema, complicated with varicose veins, freely with Richardson's styptic colloid, allowed to evaporate to half its bulk before being used; the film left by the colloid dries and contracts; it is then to be covered with a sheet of thin vulcanized india-rubber, and bandaged. Thus pressure is applied, and the atmosphere excluded.—*Journal of Cutaneous Medicine.*

ULCERS A CAUSE OF BRIGHT'S DISEASE.—Professor Fischer, of Breslau, has shown that chronic ulcers of the legs, if allowed to persist unhealed, invariably lead to amyloid degeneration of the kidney. Hence the cure of such ulcers becomes a matter of great importance. Dr. Fischer reccommends Langenbeck's continuous bath as the best treatment.—*Journal of Cutaneous Medicine.*

NEW METHOD OF REDUCING DISLOCATIONS OF THE SHOULDER.—Dr. Samuel Logan, of New Orleans, describes his method. The patient is placed on the back, while the surgeon, having removed his boots, seats himself so that his legs shall be nearly at right angles to the patient's body, but pointing rather upwards. He places the heel of one foot in the armpit, against the ribs, so as to press a little crosswise; the base of the great toe of the other foot is placed against the acromion process, and the surgeon pushes with moderate force while traction is made upon the arm.—*Transactions Am. Med. Association.*

THE INFLUENCE OF ABNORMAL MILK ON NUTRITION.—Mr. C. Meynott Tidy (*Lancet*), was recently consulted by a lady, whose infant, 6 months old, was, to use her own words, "becoming miserably smaller day by day." Undoubted signs of incipient pulmonary tuberculosis were found in the mother, and, on analyzing her milk, it was found markedly deficient in fatty matter. At Mr. Tidy's suggestion, the milk was drawn off at stated times, and, with a stated quantity of mutton suet, was fed to the child from a bottle. The child soon began to thrive astonishingly.

*Sulphate of Iron in Suppuration.*—A child burned nearly over the whole body was recently brought to the Child's Hospital at Lausanne. The suppuration was so abundant that the ward in which the patient lay become uninhabitable. The child was then placed in a bath containing two handfulls of sulphate iron. The cessation of pain was almost immediate. After repeating the bath twice a day, for fifteen or twenty minutes at a time, the suppuration moderated, the fœtid odor disappeared, and the patient rapidly recovered.—*Bost. Jour. of Chemistry.*

*Purification of Chloral Hydrate.*—Professor F. A. Flueckiger states (*Phar. Cent Hall*) that much of the commercial chloral hydrate is impure, and unfit for use until purified. It is usually met with in irregular masses containing moisture, and having a sharp, pungent odor, indicating partial decomposition. The salt may be readily purified by dissolving it in *pure* bisulphide of carbon (soluble in forty-five parts of the menstruum at 18° C.) and allowing the salt to recystallize by the spontaneous evaporation of the solvent. The product appears in colorless prismatic crystals, not hygroscopic, and free from acid reaction.

*Chloral in Midwifery.*—1. Cloral is an agent of great value in the relief of pain during parturition.

2. It may be administered under favorable circumstances during and at the close of the second stage, with the result of producing absolute unconsciousness in the same sense in which we understand unconsciousness under chloroform.

3. When thus given successfully, it has this advantage over chloroform, that it requires no interference with the patient.

4. It is desirable to retain chloroform in the position which it at present occupies in midwifery, and to reserve for the agency of Chloral the first stage of labor. If, however, Chloral or some agent having analogous properties is found successfully to relieve the pain of uterine contraction, the use of chloroform will be restricted to a lesser period of the duration of labor, or to the facilitation of manual or instrumental interference.

5. It is demonstrated that a labor can be conducted from its commencement to its termination, without any consciousness on the part of the patient, under the sole influence of Chloral.

6. The exhibition of Chloral in nowise interferes with the exhibition of chloroform.

6. The proper mode of exhibiting Chloral is in fractional doses of grs. xv. every quarter of an hour until some effect is produced; and according to the nature of that effect the further administration is to be regulated. Some patients will require doses of ʒ i, ;* and it is better to produce an anæsthetic effect by ʒ iii., given in the space of two hours, than by ʒ i., given singly.

8. The effects of Chloral are continued beyond the period of completed parturition, and the repose experienced by the patient after her labor is one of the favorable circumstances to be noted in considering its application to childbirth.

9. Any stimulating effects, in the form of general excitability, occasionally observed during the administration, have passed away very rapidly.

10. Chloral not only does not suspend, but rather promotes uterine contraction by suspending all reflex actions which tend to counteract the excitability of the centres of organic motion.

11. Labors under Chloral will probably be found to be of shorter duration than when natural, for unconscious contractions appear to have more potent effects than those which are accompanied by sensations of pain.

12. Experiment are required in order to determine whether there exists the same antagonism between Ergot and Chloral as is known to exist between Strychnia and Chloral.

---

* This dose is too large. Much of the Choral of the shops is impure, and requires great caution in its administration.—[Editors.

13. The general conditions under which Chloral is to be administered are the same as those which regulate the administration of chloroform, and the rules laid down by Sir James Simpson in connection with this subject must be rigidly adhered to.—*Edinburgh Medical Journal.*

*Antiphlogistic Value of Ergot.*—Dr. Jacobi holds that Ergot acts through the nervous system, and especially through the sympathetic, upon the unstriped muscular tissue under its control. Thus it is that Ergot produces its peculiar effect upon the muscular tissue of the uterus, the unstriped fibres of the bladder, the muscular layers of the intestines, and especially upon the muscular coat of the blood vessels. Its power in diminishing the size of the blood vessels is manifest from its value as a hæmostatic; and it is of this power we have to speak in considering its antiphlogistic effect.

This effect is noticeable in fevers generally, and particularly in fevers of the intermittent type. It is an established fact, in my opinion, that many cases of obstinate intermittent fever will, when no longer benefitted by Quinine or Arsenic, still be benefitted by the action of Ergot.

Dr. Jacobi states that he has given a mild preparation of Ergot with a uniformly beneficial effect in many cases, not uncommon in women and children, of spinal miningitis resulting in pain, slight fever, occasional convulsions, partial or total paralysis. He has given it advantageously in the first stage of the so-called infantile or dental paralysis dependent upon congestion of the spinal cord. The persistence of the disease he attributes to dilatation of the blood vessels, and he gives the Ergot to effect their contraction. In cases of chorea minor, not connected with rheumatism, but with pain in some portion of the spinal column, Ergot is often serviceable in removing the symptoms.

Dr. Jacobi uses large doses, as two drachms (to a child) of Squibb's fluid extract, in the day, or from four to seven grains of Bonjean's ergotine. When its use is to be long continued, for chronic hemorrhages, etc., he gives commonly about a scruple of the ergotine daily to an adult, and to a child in proportion to age. He has never seen any of the symptoms of so-called ergotism, whether of the spasmodic or gangrenous form, nor has he any fear of it resulting from the use of ergot in this dose.—*Medical Record.*

*Management of Scabies.*—Dr. M'Call Anderson (*Richardson's Hand-Book of Microscopy*) greatly prefers, in the treatment of scabies, in private practice, the following formula: ℞ Styracis liquidi, ℥j.; adipsis, ℥ij. Melt and strain. This is a clean-looking ointment, has a pleasant aroma, kills the acari, and does not irritate the skin in the least, but, on the contrary, rather soothes it.

Puncture of the Abdomen for the Relief of Tympanitis.—The Dublin Quar. Jour. of Med. Science mentions three cases in which marked relief was afforded by this operation. In one the distension was caused by the pressure of an ovarian tumor on the intestine. The puncture was made in the cœcal region, and was repeated daily, more than fifty times, at the request of the patient. At the autopsy no traces of the punctures could be observed. The second case was that of a man 61 years old. Eight punctures were made in fourteen days, with great relief and no unpleasant results. In another case, reported in the Practitioner, the operation was performed upon a patient with double pneumonia. The punctures, two in number, were made over the transverse and descending colon, and gave great relief. The patient, however, died of pneumonia, but no traces of the puncture could be found after death, except on the surface of the body. The instrument used was an exploring trochar (m. I Weiss.)

Chloral in Delirium Tremens.—In the Centralblatt fur die Med. Wissen. Dr. Curschmann relates some experiments in the treatment of delirium tremens by chloral hydrate. There were twenty four males thus treated from the age of twenty-four to fifty. Two cases were complicated by pneumonia, four with surgical diseases, and the rest without complication. All were treated by chloral hydrate in wine, and in two instances by subcutaneous injection, which is not to be recommended. Clysters cannot be used in D. T. The dose given was 3 to 4 grammess—one drachm, at first, afterwards more. The smallest quantity which caused sleep was five grammes, but one patient had as much as 25 grammes in 22 hours. The remedy was more successful in beer drinkers than in spirit-drinkers. The pulse and respirations frequently sank soon after the administration of the drug. The duration of the sleep was 8 to 21 hours, and the patients awoke cured. He considers that the cure by chloral is much mere rapid in D. T. than when any other remedy is made use of.

The Local and Superficial Anæsthetic Effects of the Phenols.—According to Dr. Edward R. Squibb, of Brooklyn, N. Y. Med. Journal, the best of these is cresol or cresylic acid, and next phenol or the crystalized carbolic acid. But practically the cheap mixture of the two, called coal-tar creosote or impure carbolic acid, is as good as either. The prompt and complete effect of very dilute aqueous solutions of this creasote upon the pain of burns, erysipelas, etc., led him to infer peculiar anæsthetic properties many years ago, and the numbness or insensibility produced upon the hands by handling it confirmed the idea.

*On the Removal of Subcutaneous Tumors without Hemorrhage or Loss of Skin.*—Mr. Henry Lee read a paper on the above subject before the Royal Medical and Chirurgical Society. He stated that he had been in the habit for some time of removing small tumors by India-rubber thread. He found that the pressure of the thread would rapidly, by a process of linear mortification or of ulceration, cut through the base of a tumor. This principle might be applied to the surface as well as to the base of any growth that might have to be removed, and was peculiarly applicable to vascular tumors of the neck and face. A crucial line of ulceration was first made through the skin by the continued pressure of India-rubber bands or thread. Needles were then inserted below the flaps of skin thus produced, and the skin was dissected back, from the centre towards the circumference, by the pressure of the India-rubber. The base of the tumor was then cut through in the same way, so that the whole of it was enucleated without the aid of the knife. The process went on much more rapidly than might be expected, and it was comparatively safe, as the India-rubber thread always, on account of its elasticity, remained tight. The circulation could not cansequently be re-established in a part once strangulated, and so far the danger of blood-poisoning was avoided. *Brit. Med. Journ.*

*Aconite is the Child's Remedy.*—We don't mean to say that it is not a remedy for the adult, but it is especially the remedy for the acute diseases of childhood. In fever or slight inflammation, the result of cold, it is the only remedy required. In infantile remittent fever, it prepares the patient for the use of diaphoretics, and diuretics, and when from malaria, the administration of Quinine. If employed early it will arrest a bronchitis or pneumonitis, as it will a tonsilitis, sore thoat, or mucous croup. It will relieve gastric irritation, cure diarrhœa the result of irritation or inflammation of the intestines, or dysentery, when not epidemic. We employ it alike in all cases where there is frequency of the pulse, increase of temperature, irritation of the cerebro-spinal centers, or local disease with determination of blood, or inflammation in its first or active stage.—*Eclectic Med. Journal.*

*Shampoo, for Baldness.*—Take of rum 500 parts, alcohol 75, distilled water 75, Tincture of Cantharides 3, Carbonate of Ammonia 3, Carbonate of potassa 3 ; mix. First wash the head with warm water, dry it, and then rub on some of the above preparation for several minutes.

*Tooth Ache.*—Take of Cloroform 5, Sydenham's Laudanum 2, and tincture of benzoin 10 parts ; mix. Apply it to the cavity of the carious tooth on a piece of cotton, and renew it from time to time.

